# Atrioventricular Node Dysfunction in Heart Failure: New Horizons from Pathophysiology to Therapeutic Perspectives

**DOI:** 10.3390/jcdd12080310

**Published:** 2025-08-15

**Authors:** Isabella Fumarulo, Elia Nunzio Maria Salerno, Andrea De Prisco, Salvatore Emanuele Ravenna, Maria Chiara Grimaldi, Francesco Burzotta, Nadia Aspromonte

**Affiliations:** 1 Department of Cardiovascular Sciences, Fondazione Policlinico Universitario A. Gemelli IRCCS, 00168 Rome, Italy; isabella.fumarulo@guest.policlinicogemelli.it (I.F.); elianunziomaria.salerno01@icatt.it (E.N.M.S.);; 2 Department of Cardiovascular Sciences, Catholic University of the Sacred Heart, 00168 Rome, Italy; 3 Department of Life Science, Health and Health Professions, Università degli Studi Link, 00165 Rome, Italy

**Keywords:** heart failure, atrioventricular node, cardiac resynchronization therapy

## Abstract

Heart failure (HF) is characterized by adverse myocardial remodeling involving both the contractile cardiomyocytes and the conduction tissue. HF is often associated with atrioventricular (AV) node dysfunction, which frequently leads to conduction delays and subsequent dyssynchrony ultimately related to adverse clinical outcomes. Despite its clinical relevance, AV node pathology in HF remains poorly explored. This review aims to investigate the pathophysiology underlying AV node dysfunction and the clinical implications for patients with HF, and to provide an overview of current therapeutic approaches, including an analysis of potential future treatments.

## 1. Introduction

Heart failure is a complex, multisystemic syndrome with an increasing prevalence worldwide [1], burdened by significant morbidity and mortality, poor quality of life, and high costs related to frequent hospitalizations and the need for advanced therapies [2]. HF is characterized by the adverse remodeling of the myocardium, with volumetric dilation and worsening of contractility [3,4], supported by the dysfunctional vicious circle of neurohormonal alterations typical of this pathology. In this context, the conduction system is also likely affected, undergoing a negative remodeling process as well, which leads to electrical issues and further worsening of cardiac function.

Atrioventricular (AV) node dysfunction is quite frequent in patients affected by HF. The prevalence of first-degree AV block (AVB) jumps from 4% in the general population, to a rate between 15% and 51% in HF patients [5], associated with an increased risk of mortality and hospitalizations. First or second-degree AVB is an independent risk factor in idiopathic dilated cardiomyopathy, especially if associated with either reduced left ventricular ejection fraction (LVEF) or frequent ventricular couplets [6].

Despite this relevant data, the mechanisms underlying AV node dysfunction in HF are not well understood and there are no clear indications about specific treatment.

Cardiac resynchronization therapy (CRT) may result in better outcomes in HF patients, through the optimization of AV delay. A better understanding of the pathophysiological basis of AV node dysfunction in HF may lead to the development of targeted therapeutic strategies to improve symptoms and overall survival, prior to device therapy. In the next paragraphs, we explore AV node pathology in HF, and clinical implications, current and potential therapeutic options.

## 2. Physiology of the Atrioventricular Node

The AV node is one of the key structures in the heart’s electrical conduction system playing a crucial role in transmitting and coordinating the electrical impulses that determine the heart’s rhythm.

It consists of a small group of highly specialized cells located in the inferior–posterior region of the interatrial septum, close to the opening of the coronary sinus and above the fibrous ring separating the atria from the ventricles. In a right-dominant heart, the AV node is supplied by the right coronary artery.

The AV node’s strategic position allows it to receive the impulse generated by the sinoatrial (SA) node and transmit it to the bundle of His and, thereafter, to the ventricular conduction network and myocytes. The delay in conduction between the atria and ventricles at the AV node is physiologically important.

This highly specialized conducting tissue has distinctive electrophysiological properties: automaticity, slow conduction, and a prolonged refractory period.

Although the SA node is the main pacemaker (PM), AV node cells can spontaneously generate electrical impulses under certain conditions, such as SA node blocks or slower activity. The ultrastructure of these cells, the specific ionic mechanisms, and their high intercellular resistance explain the slow passage of the impulse through the node. Moreover, the AV node has a longer refractory period compared to other heart conduction system structures. The conduction velocity through the AV node is indeed slower (about 0.05 m/s) compared to that of the atrial fibers (about 1 m/s). The total delay imposed by the AV node is 0.09–0.12 s. The intrinsic rate of the AV node is 40 to 60 beats per minute (bpm).

First, it allows the complete atrial depolarization, contraction, and emptying of atrial blood into the ventricles prior to ventricular depolarization and contraction, improving pump efficiency and cardiac hemodynamics. Second, the low conduction velocity protects the ventricles from extremely high rates in the event of atrial tachyarrhythmias.

Indeed, the activity of the AV node is strongly modulated by the autonomic nervous system: the sympathetic beta-adrenergic receptors activation increases conduction velocity and shortens the refractory period; the parasympathetic system, through the vagus nerve, slows conduction and increases the refractory period. This regulation allows the AV node to adapt impulse flow according to the body’s needs, such as during physical exercise or at rest [7,8].

## 3. Pathophysiology of AV Node Dysfunction in HF

Although detailed electrophysiological data in humans are lacking on this topic, some evidence from animal models allows us to hypothesize the causes underlying cardiac conduction system dysfunction in HF.

Verkerk et al. analyzed the electrical properties of SA node cells from rabbits with volume and pressure overload-induced HF and the patch clamp technique [9]. They found an increased intrinsic cycle length and decreased diastolic depolarization rate in HF subjects, with reduction of the hyperpolarization-activated “pacemaker” current (If) and a slow component of the delayed rectifier current (IKs).

Other recent studies have investigated the properties of the AV node more specifically in the context of HF with genetic analyses.

Yanni et al. investigated the changes in ion channel gene expression in ischemic induced HF in rats [10]. After the ischemic trigger (ligation of the proximal left coronary artery), the authors found a significant increase in left ventricular (LV) diastolic pressure and SA node dysfunction, with a reduced intrinsic heart rate and an increased corrected SA node recovery time. There was also evidence of AV dysfunction with the prolongation of the PQ interval. The investigators demonstrated that clinical alterations were related to modified expression of several genes involved in the regulation of ion channels, gap junction channels, calcium (Ca^2+^), sodium (Na^+^), and hydrogen (H^+^) exchangers, and several receptors. Therefore, the elevated LV diastolic pressure, typical of HF, causes an extensive remodeling of ion channels, gap junction channels, exchanger proteins, and receptors in the conduction cells, finally leading to dysfunction of both SA and AV nodes.

Wilson et al. recently investigated the changes in the nodal transcriptome in a murine model of pressure overload-induced HF caused by transverse aortic constriction [11]. They found significant changes in 5.6% of the transcriptome, by RNA sequencing, mostly in a proinflammatory upregulation. The authors found the downregulation of Na^+^, Ca^2+^, and K^+^ channel transcripts and significant changes in transcripts involved in the modulation of the PR interval, in transcripts responsible for the metabolism of the sarcomere, and in several pathways involved in cardiac remodeling. The immune system was found particularly activated, with evidence of the proliferation and infiltration of inflammatory cells in the AV node, enhancing inflammation and fibrosis. Thus, pressure overload-induced HF leads to remodeling of the sarcomere (shifting from fatty acid to glucose metabolism), to activation of proinflammatory pathways, to downregulation of several ion channels, and finally to a significant increase in the PR interval.

Therefore, although similar studies performed on human subjects are lacking, this preclinical evidence focuses on the principal mechanisms underlying the AV node dysfunction in HF.

HF is due to the heart’s inability to pump enough blood for the body’s needs; this results in the activation of multiple compensatory mechanisms (fluid retention, sympathetic activation, vasoconstriction) to increase cardiac output, leading to a significant increase in intracardiac pressures [12]. Prolonged high intracardiac pressures function as a stimulus for mechanical and genetic changes, both in “working” cardiomyocytes (responsible for heart contractility and relaxation) and in “conduction” cardiomyocytes (responsible for the cardiac conduction system). The genetic modulation involves several proteins, channels, ion exchangers, and receptors, resulting in the perturbation of normal cardiac electrophysiology and haemodynamics.

**Figure 1:**
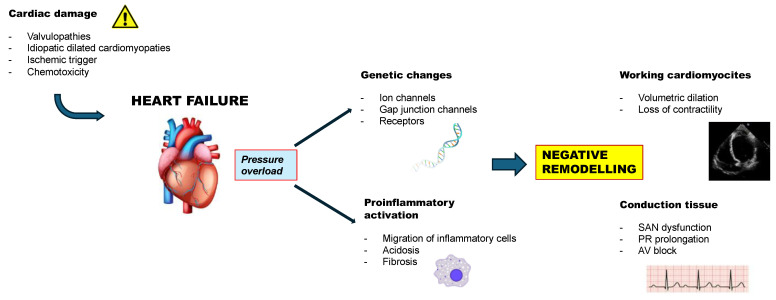
Pathophysiology of AV node dysfunction in HF. AV: atrioventricular; HF: heart failure; SAN: sinoatrial node.

Furthermore, the constant pressure overload on cardiomyocytes activates some proinflammatory pathways, promotes the migration of inflammatory cells, and activates reactions that culminate in fibrosis. This leads to both loss of contractility and issues in the cardiac conduction system. The proinflammatory environment can lead to acidosis, which can decrease L-type Ca^2+^ current (I(Ca,L)) and rapid delayed rectifier current (I(Kr)), affecting the AV node function [13] (Figure 1).

## 4. Clinical Implications of AV Node Dysfunction in HF

The function of the AV node can be easily accessed through the 12-leads electrocardiogram (EKG), in particular with the evaluation of the PR interval. This interval increases progressively with age and body mass index (BMI) and is physiologically longer in men compared with women. Autonomic as well as structural cardiac abnormalities may lead to prolongation of the PR interval. In the elderly, the responses to catecholaminergic or inotropic stimuli are blunted; fibrosis and calcification of the cardiac skeleton could also lead to delays in electrical conduction.

The analysis of data from the Framingham study showed that 1.6% of the general population had a PR interval > 200 ms and that first-degree AVB was associated with an increased risk of all-cause mortality, atrial fibrillation (AF), and pacemaker insertion at 20 years’ follow-up [14]. However, reports from the Third National Health and Nutrition Examination Survey (NHANES-III) [15,16], and the Finnish Social Insurance Institution’s Coronary Heart Disease Study (CHD Study) [17] have shown no significant associations between prolonged PR interval and all-cause mortality.

Some data, on the contrary, revealed that only a short PR interval (<120 ms) was associated with increased all-cause mortality. However, a greater contribution of the P wave duration to the PR interval (P-wave duration to PR interval ratio > 0.7) was associated with increased mortality both in the short and long PR interval groups [18].

The ABC (Health, Aging and Body Composition) study among 2722 patients aged 70 to 79 with no functional disability found a significant association between increasing baseline PR interval and increasing risk of incident HF and AF at 10 years. PR interval duration did not, however, affect 10-year all-cause mortality. The prevalence of first-degree AVB in the ABC study population was 12%, higher than in other studies, but this difference could reflect the participants’ older age at baseline (mean age 74 years) [19].

HF is associated with widespread electrophysiological remodeling of the cardiac conduction system, for the reasons mentioned above, potentially resulting in prolongation of the PR and QRS intervals, reduced RR variability, and AF.

Animal models showed a huge modulation of ionic channels that occurs in every stage of cardiac failure [10,11] and bradycardia and AV electrical abnormalities are common in HF patients. Beta blockers represent a fundamental pillar in the treatment of these patients, but their use could be associated with AV blocks, due to their negative dromotropic effect, primarily shown at the level of the AV node [20].

Both in patients with underlying ischemic heart disease and those with non-ischemic cardiomyopathy, approximately one-half of arrhythmic deaths are probably bradycardic in origin, including due to high-degree AVB.

In the Korean Heart Failure registry, the prevalence of first-degree AVB (PR > 200 ms) was about 10% among patients presenting with acute heart failure. First-degree AVB in combination with a long QRS predicted in-hospital cardiac death and all-cause mortality [21].

The MADIT-CRT trial (Multicenter Automatic Defibrillator Implantation Trial With Cardiac Resynchronization Therapy) enrolled patients without left bundle-branch-block (LBBB) who were randomized to the control arm, undergoing implantable cardioverter defibrillator (ICD) therapy or CRT-D (undergoing cardiac resynchronization therapy). In the subgroup of patients with a prolonged PR interval (>230 ms), CRT-D treatment was associated with a 73% reduction in the risk of HF or death and an 81% decrease in the risk of all-cause mortality compared with ICD only [22]. The excessive prolongation of the interval between atrial and ventricular contractions can adversely impact cardiac function through suboptimal ventricular filling: the passive ventricular filling phase can be fused with or prematurely interrupted by atrial contraction. There could also be diastolic mitral regurgitation: the early atrial relaxation, which results in a drop in atrial pressure, occurs before papillary muscle contraction, which closes the mitral valve. The restoration of the physiological AV sequence may improve LV diastolic filling and abolish presystolic mitral regurgitation.

An analysis of the COMPANION trial showed that 50% of patients eligible for CRT had first-degree AVB (PR > 200 ms). Patients in the prolonged PR interval group were more likely to have ischemic cardiomyopathy, a wider QRS complex, and AF. A PR interval > 200 ms in the group assigned to medical therapy was associated with a 41% increased risk of all-cause mortality and HF hospitalization, while no significant difference was found for patients with first-degree AVB assigned to CRT [23].

In the CARE-HF study, a PR interval > 200 ms at baseline predicted an unfavorable outcome in patients with chronic HF. The PR interval, measured at three months after randomization, remained a strong predictor of adverse outcomes (all-cause mortality and HF hospitalization) in the single- and multiple-variable analyses after inclusion of CRT in the model [24].

Whether first-degree AVB is a marker of subclinical coronary artery disease remains controversial. The CARISMA (Cardiac Arrhythmias and Risk Stratification After Acute Myocardial Infarction) trial enrolled 297 patients after acute myocardial infarction with ejection fraction (EF) < 40%, who underwent a loop recorder implantation to assess arrhythmic burden. High-degree AVB was documented in 29 patients; the total number of high-degree AV block episodes was 124, corresponding to 14% of all arrhythmias, but only 20 (42%) were symptomatic. One third of patients developed AF. In this study, AVB was the strongest predictor of all-cause mortality and cardiac death [25].

All these studies have shown that AV node dysfunction is quite frequent in patients affected by HF, with significant implications in terms of worse clinical outcomes.

## 5. Therapeutic Strategies

For a long time, the AV node has received limited attention as a direct therapeutic target in HF management. The AV node has been traditionally considered as a passive relay point, and the technical challenge of selectively modulating AV nodal conduction without impairing AV synchrony or inducing bradyarrhythmias, particularly in patients in sinus rhythm, limited the therapeutic approaches. Moreover, in the pre-device era, therapeutic options for safely manipulating AV nodal function were scarce. For example, in HF patients with prolonged PR interval but preserved rate control, attempts to modify conduction could risk worsening cardiac output, whereas in selected patients with marked conduction delay, careful modulation could improve diastolic filling and overall hemodynamics.

### 5.1. Pharmacological Therapy

The AV node is often the target of pharmacological and non-pharmacological interventions aimed at modulating heart rate and conduction. Medications that affect the AV node include beta blockers, which decrease beta-1 adrenoceptor activity; non-dihydropyridine calcium channel blockers, which inhibit Ca^2+^ currents and reduce AV conduction; digitalis, which enhances vagal tone and slows AV node conduction, a valid option for rate control particularly in heart failure with preserved ejection fraction (HFpEF) patients with atrial fibrillation and a need to avoid hypotension; and adenosine, which produces inhibitory effects on the AV node.

Moreover, sympathomimetic agents such as epinephrine or isoproterenol increase heart rate by stimulating the AV node. Indeed, vagolytic agents such as atropine block the effects of the vagus nerve, which increase heart rate and AV conduction. Also, ivabradine have an inhibitory effect on the AV node, primarily by slowing conduction through the node, targeting the I_f_ currents, and has shown utility in reducing HF hospitalizations in selected patients.

Management of AV node dysfunction occurrence in HF patients is an ordinary challenge for cardiologists. Beta blockers and other rate-reducing drugs are some of the cornerstones of HF therapy [26], important to reduce symptoms, hospitalizations, and mortality, but their use may lead to the development of reversible AV conduction disturbance.

According to the European Society of Cardiology (ESC) guidelines [26], reversible AV node dysfunction is not an indication for permanent pacing, and it often leads clinicians to discontinue rate-reducing therapy, which may affect the prognosis of HF patients. Furthermore, a direct cause-and-effect relationship between drugs and AVB is not so clear. Several studies have demonstrated frequent relapse of AVB after beta blocker discontinuation [27,28], showing that true drug-related AV block was a minority of cases [29].

Moreover, a high rate of PM implantation has been documented during follow-up of patients with “reversible AV” block, despite the discontinuation of rate-reducing drugs [28,30]. The use of beta blockers unmasks a subclinical disorder of the conduction system that would have manifested itself over time anyway. This data may suggest that withholding beta blockers may not benefit HF patients who develop AV block, especially if it is a low-grade block, and an early device therapy may allow HF patients to maintain the benefit of beta blockers therapy.

According to this concept, the American Guidelines, unlike the European ones, suggest pacing therapy in patients who require chronic administration of beta blockers, also without further observation for drug washout or reversibility (class IIa recommendation, level of evidence B) [31].

### 5.2. Device Therapy

It is common for patients with HF to require device implantation over the course of their disease.

According to the ESC guidelines, ICD implantation is recommended for HF patients with severely reduced LVEF (less than 35%), which does not improve after 3 months of optimized guideline-directed medical therapy (GDMT) for primary prevention of sudden cardiac death (SCD) [26].

Patients with HF (especially those with EF < 35%) often develop high-grade intraventricular conduction delays with specific electrocardiographic changes such as QRS prolongation more than 120 ms and left bundle-branch-block (LBBB). In addition, in more than 50% of patients, HF can lead to a prolonged PR duration [32]. Therefore, HF is often associated with AV and interventricular conduction issues, promoting contractile dyssynchrony and further worsening of cardiac systolic performance.

In this regard, resynchronization therapy may have a significant role in HF with reduced ejection fraction (HFrEF) and AV dysfunction. The development of AVB may accelerate the progression of the disease toward device implantation.

One of the main problems of ventricular stimulation is the development of a contractile dyssynergy, that may worsen EF, inducing the PM-induced cardiomyopathy [33], especially in case of high percentage of ventricular stimulation, as in AV block. In detail, some trials demonstrated that apical positioning of the PM catheter is associated with a worse deterioration of LVEF compared with non-apical positioning [34,35]. In line with this, trials on HFrEF patients with a conventional indication to pacing therapy showed a superiority of resynchronization therapy on right ventricular pacing. Many trials supporting the use of CRT in patients with HF were conducted in sinus rhythm (SR). For this reason, the latest ESC guidelines for cardiac pacing suggest the use of CRT in symptomatic HF patients in SR with LVEF < 35% and a QRS duration > 130 ms [26].

The indications for CRT in patients with HFrEF are more consistent in those with New York Heart Association (NYHA) classification of II-IV. The sub-analysis of long-term follow-up of the MADIT-CRT trial evaluated the benefit of CRT in patients with NYHA I and chronic coronary syndrome, showing a non-significant reduction in death for any cause [36,37].

On the other hand, the CARE-HF trial enrolled 813 patients with NYHA III or IV and randomized patients with LV systolic dysfunction and cardiac dyssynchrony to cardiac resynchronization therapy against medical therapy alone. The primary endpoint was death from any cause or an unplanned hospitalization for a major adverse cardiovascular event (MACVE). After a follow-up of almost 30 months, the results have demonstrated that cardiac resynchronization reduces the interventricular mechanical delay, the end-systolic volume index, and the area of the mitral regurgitant jet, increasing the EF and reducing death or hospitalization [38].

Other studies focused on patients with moderate to severe systolic dysfunction requiring antibradycardia pacing, with less limitation about the NYHA class. In the BLOCK-HF trial (Biventricular Versus Right Ventricular Pacing in Heart Failure Patients With Atrioventricular Block), patients with HF, AVB, and NYHA class I-III were randomized to PM or CRT therapy.

The primary outcome was death from any cause, an urgent care visit for HF requiring intravenous therapy, or an increase in the left ventricular end-systolic volume index of 15% or more. The analysis showed that biventricular pacing reduced the incidence of the primary outcome, demonstrating that CRT pacing was superior to PM.

The pathophysiological reason is that RV pacing is associated with LBBB-like conduction, which results in contractile dyssynchrony with a delayed activation of the left ventricular lateral free wall and basal wall. This dyssynchronous contraction is associated with a progressive increase of ventricular filling pressures, with consequent worsening of ventricular remodeling and contractile dysfunction [33].

This evidence supports the guidelines’ recommendation of CRT implantation rather than RV pacing for patients with HFrEF, who have an indication for ventricular pacing and high-degree AVB to improve prognosis, regardless of symptoms status, in class IA [39].

Certain clinical scenarios may especially benefit from targeted AV nodal intervention. Patients with fast atrial fibrillation refractory to pharmacological rate control, or those with tachycardia-induced cardiomyopathy, may benefit from an “ablate and pace” approach, preferentially coupled with CRT to optimize synchrony. In such cases, appropriate pacing rate selection is critical to balance adequate cardiac output with avoidance of excessive myocardial workload; current guidelines recommend tailoring the lower rate limit to patient-specific haemodynamics and comorbidities.

The following table summarizes the major characteristics of the studies mentioned above (Table 1).

## 6. Future Directions

Some studies reported the role of hyperpolarization-activated cyclic nucleotide-gated channel 4 (HCN4) also in the AV node. In fact, it seems that in AV dysfunction there is a decreased expression of miRNA-encoding Ca^2+^ channels, Ca^2+^-handling molecules, HCN channels (carrying the funny current responsible for pacing), K^+^ channels, connexins, but also of the cardiac Na^+^ channel and the Na^+^/K^+^ pump. Consequently, this can reduce the density of potassium, calcium, and the funny currents.

In their study, Mesirca et al. [40] evaluated the regulatory pathway of the AV node remodeling, hypothesizing an miRNA-mediated regulatory mechanism that can repress ion channel expression. According to this idea, gene therapeutic interventions through anti-miRNAs can be a valid future alternative option to restore AV conduction in addition to other therapeutic options [41]. Further studies are needed to validate this hypothesis; nevertheless, this could become a promising field for future research.

## 7. Conclusions

HF is characterized by negative remodeling of the myocardium, involving both the contractile cardiomyocytes and the conduction tissue. This leads to genetic changes and activation of proinflammatory pathways that cause conduction issues, including AV dysfunction, with a significant impact on cardiac dyssynchrony and, consequently, on prognosis. An early PM implantation could allow for an opportunity to maintain the benefit of beta blocker therapy, a cornerstone in HF therapy. Technically, the cardiac resynchronization therapy (with the pacing on septum or through the coronary sinus), may offer better outcomes than RV apical pacing in terms of paraphysiological contractility of the LV, reverse remodeling, and prognosis. Another promising research field is miRNA: targeting these molecules, involved in multiple channels expression, could allow a better regulation of electrical current, with interesting results in managing the electrical issues related to HF.

From a translational perspective, the restoration of the AV nodal function may contribute to direct cardiomyocyte gene expression and metabolism in a virtuous direction, promoting reverse remodeling in patients with HF.

## Figures and Tables

**Table 1:** Studies about device therapy in patients affected by HF and electrical issues. CRT: cardiac resynchronization therapy; GMT: guideline-directed medical therapy; HF: heart failure; ICD: implantable cardioverter defibrillator; LVEDD: left ventricle end diastolic diameter; LVEDDi: left ventricle end diastolic diameter index; LVEF: left ventricular ejection fraction.

Study	Author	Follow Up	Population	NYHA Class	Randomization	Outcomes	Results
MADIT-CRT	Arthur J. Moss et al.	4.5 years	1820 pts, LVEF ≤ 30%, QRS ≥ 130 ms	I–II	CRT-D vs. ICD alone (3:2)	Death from any cause or a nonfatal HF event	CRT-D vs. ICD alone (17.2% vs. 25.3%), [HR 0.66; 95% CI, 0.52 to 0.84; ***p* = 0.001**]
MADIT-CRT (Long term)	Emily P. Zeitler	7 years	1820 pts, LVEF ≤ 30%, QRS ≥ 130 ms	I–II	CRT-D vs. ICD alone (3:2)	Death from any cause or a nonfatal HF event	CRT-D vs. ICD alone [HR 0.66, 95% CI, 0.30 to 1.42; ***p* = 0.29**].
CARE-HF	John G.F. Cleland et al.	29.4 months	813 pts, LVEF ≤ 35%, LVEDDi ≥ 30 mm, QRS ≥ 120 ms	III–IV	GMT + CRT vs. GMT alone (1:1)	Death from any cause or an unplanned hospitalization for a MACVE	CRT vs. GMT alone (39% vs. 55%), [HR 0.63; 95% CI, 0.51 to 0.77; ***p* < 0.001**]
MIRACLE	William T. Abraham et al.	6 months	453 pts, LVEF ≤ 35%, QRS ≥ 130 ms, LVEDD ≥ 55 mm, 6MWD ≥ 450 mt	III–IV	GMT + CRT vs. GMT alone (1:1)	Improvement of NYHA functional class, quality of life and the distance walked in six minutes.	GMT + CRT vs. GMT alone: distance walked in six minutes (+39 vs. +10 m, ***p* = 0.005**), changes of NYHA classes (***p* < 0.001**), quality of life (18.0 vs. 9.0 points, ***p* = 0.001**), time on the treadmill during exercise testing (+81 vs. +19 s, ***p* = 0.001**), and ejection fraction (+4.6 percent vs.. 0.2 percent, ***p* < 0.001**)
MUSTIC	Serge Cazeau et al.	6 months	67 pts, LVEF ≤ 35%, QRS ≥ 150 ms, LVEDD ≥ 60 mm	III	All received atrio-biventricular pacing. Inactivated pacing vs. Atrio-biventricular pacing	Distance walked in six minutes, quality of life, peak oxygen consumption, hospitalizations related to HF	Atrio-biventricular pacing vs. inactivated pacing (399 ± 100 m vs. 326 ± 134 m, ***p* < 0.001**); (−31%, ***p* < 0.001**); (−8%, ***p* < 0.03**); (−52/3, ***p* < 0.05**)
COMPANION	Michael R. Bristow et al.	2 years	1520 pts, LVEF ≤ 35%, QRS ≥ 120 ms, PR > 150 ms	III-IV	GMT vs. GMT + CRTP vs. GMT + CRT-D (1:2:2)	Time to death from or hospitalization for any cause	CRTP + GMT vs. GMT alone (HR 0.81; ***p* = 0.014**); CRTD + GMT vs. GMT alone (HR, 0.80; ***p* = 0.01**)
PATH-CHF II	Christoph Stellbrink et al.	1 year	77 pts, LVEF ≤ 35%, QRS ≥ 120 ms, PR > 150 ms	II-> IV	ICD vs. ICD + Pacing	Improvement in functional capacity	ICD + Pacing vs. ICD alone (Difference of 12.5% in the primary endpoints with 80% power, ***p* < 0.05**)

## Data Availability

There is no new data associated with this article.
